# Molecular signatures and biomarker development for limbic-predominant age-related TDP-43 encephalopathy (LATE)

**DOI:** 10.1007/s00401-026-03027-0

**Published:** 2026-05-15

**Authors:** Ling Wu, Tobilola Akingbade, Peter T. Nelson, Shih-Hsiu J. Wang, Bin Xu

**Affiliations:** 1 Biomanufacturing Research Institute and Technology Enterprise (BRITE), North Carolina Central University, Durham, NC USA; 2 Duke-UNC Alzheimer’s Disease Research Center, Durham, NC USA; 3 Department of Pharmaceutical Sciences, North Carolina Central University, Durham, NC USA; 4 Department of Pathology, University of Kentucky, Lexington, KY USA; 5 Sanders-Brown Center On Aging, University of Kentucky, Lexington, KY USA; 6 Departments of Pathology and Neurology, Duke University Medical Center, Durham, NC USA

**Keywords:** LATE, Molecular signature, Biomarkers, Biofluids, TDP-43, Tau

## Abstract

Limbic-predominant age-related TDP-43 encephalopathy (LATE) is a neurodegenerative disease marked by TDP-43 proteinopathy, affecting approximately one-third of individuals aged 80 and above. LATE neuropathological change (LATE-NC) is characterized by the accumulation of phosphorylated TDP-43 preferentially in the limbic system, with potential extension to the neocortex and other brain regions. Notably, the anatomic pattern of LATE-NC differs from that seen in frontotemporal lobar degeneration with TDP-43-immunoreactive inclusions (FTLD-TDP).  LATE-NC can occur in a “pure” form but more commonly exists alongside other dementia-related comorbidities, including both degenerative and vascular pathologies. When those “mixed” pathologies are factored in, LATE contributes significantly to cognitive decline in human populations.  However, LATE currently lacks a molecular-specific diagnostic method for definitive diagnosis in living people. There are new consensus-based guidelines for predicting the presence of either pure LATE-NC or LATE-NC combined with Alzheimer’s disease neuropathologic change (ADNC). Aimed at developing more specific diagnostic methods, recent research efforts have been directed toward identifying unique features on neuroimaging and molecular signatures in biological fluids such as blood and cerebrospinal fluid to facilitate clinical diagnosis for LATE. This review discusses current progress in molecular understanding of LATE-NC, the search for biomarkers for LATE, and highlights key gaps that need to be addressed to advance early detection and improve patient management and clinical trial stratification.

## Introduction

Alzheimer’s disease (AD) for many years was thought to be virtually synonymous with the concept of clinical amnestic dementia, and many studies have been carried out to understand key hallmarks of AD neuropathologic change (ADNC) (amyloid-beta (Aβ) plaques and tau neurofibrillary tangles (NFTs)) [66, 100, 157]. However, over the past several decades, autopsy studies showed that a substantial number of patients diagnosed clinically as AD dementia do not show sufficient ADNC at autopsy to explain their symptoms, highlighting the role of other brain pathologies in dementia [94]. These findings were further supported by results from patients clinically diagnosed with AD, who did not respond well to therapies and/or who had negative results for amyloid beta imaging [100].

In 2019, limbic predominant age-related TDP-43 encephalopathy (LATE) was formally recognized as a neurodegenerative disease that commonly affects the brains of patients aged 80 years and above and can potentially worsen cognitive impairment as a “pure” entity or in combination with other neurodegenerative diseases [98]. The neuropathological findings of this disease were termed LATE neuropathological change (LATE-NC), characterized by the accumulation of TDP-43 aggregates particularly in the limbic brain regions, especially in the amygdala and hippocampus [56, 92, 98]. Thus in this review, we use AD and LATE to refer to the different disease entities that contribute to amnestic dementia, and use ADNC (characterized by Aβ plaques and NFTs) [13, 47, 89, 90, 132] and LATE-NC (characterized by TDP-43 protein aggregates with a limbic-predominant distribution) [98], respectively, to refer to the underlying neuropathology.

TDP-43 is a 43 kDa transactive response (TAR) DNA-binding protein that is normally localized in cell nuclei. In diseases with TDP-43 pathology, TDP-43 becomes mislocalized or aggregated in the cytoplasm which leads to loss of its nuclear functions and gains of deleterious functions [73, 98]. The accumulation of phosphorylated TDP-43 aggregates in the cytoplasm and cellular processes of neurons and glial cells is known as TDP-43 proteinopathy [4, 28, 57]. In addition to the limbic structures, abnormal accumulation of TDP-43 has also been seen in the olfactory bulb, neocortex, basal ganglia, and more scarcely in the brainstem of some LATE-NC patients [56, 98].

TDP-43 proteinopathy has long been recognized as the pathological hallmark in the majority of amyotrophic lateral sclerosis (ALS), a subset of frontotemporal lobar degeneration (FTLD) [72, 80, 104], and several dozen other neurological diseases [22]. With regard to amnestic dementia, TDP-43 proteinopathy was first described in a subset of patients with comorbid ADNC and/or hippocampal sclerosis (defined as neuronal loss and gliosis in the CA1 and subiculum out of proportion to the degree of ADNC) [4]. Over the years, TDP-43 proteinopathy in elderly non-ALS, non-FTLD individuals with cognitive impairment was often studied and many different terminologies were used to describe this pathology, such as hippocampal sclerosis dementia, cerebral age-related TDP-43 with sclerosis, AD + TDP-43, and TDP-43 pathologies in the elderly [76, 96, 97]. However, it is increasingly recognized that many patients with limbic-predominant TDP-43 proteinopathy do not necessarily have hippocampal sclerosis or significant ADNC. As such, unlike the pathological hallmarks of Aβ plaques and NFTs, TDP-43 proteinopathy is not a feature of ADNC per se. The LATE-NC working group report brought together a consensus group in 2019 to provide routine diagnostic guidelines, disambiguating the concept, and has now been cited > 1600 times [98]. The designation of LATE has brought attention to the distinct contributions of limbic-predominant TDP-43 proteinopathy to dementia and led the field to focus on understanding the etiology, clinical presentation, and imaging features of this disease entity.

ADNC and LATE-NC rank among the most common pathologies with significant association with dementia, and both present with similar memory-related impairment, which often leads to misdiagnosis [12, 98, 99, 118, 149, 153]. Approximately one in three individuals who reach the age of 85 will develop LATE-NC in their lifetime [99], and studies have revealed that about 40% of cognitive decline is closely associated with ADNC and about 20% can be attributed to LATE-NC [98, 102]. In the “oldest-old”, the attributable risk of LATE-NC has been shown to be even greater than ADNC in several high-quality community-based cohorts [88, 118].

Autopsy studies of patients with dementia have revealed that mixed pathologies, signifying a combination of protein-aggregating and cerebrovascular pathologies, are very common and are the rule rather than the exception in aging brains with cognitive impairment [59, 61, 113, 114, 116, 121]. Therefore, it is not surprising that LATE-NC often coexists with other neurodegenerative diseases, including ADNC and Lewy body disease (LBD) [1, 100, 140, 145]. While LATE-NC is strongly associated with other neurodegenerative pathologies such as ADNC and LBD, LATE-NC often exists independently of ADNC [38, 41, 66, 108, 145]. LATE-NC with no or low level ADNC [47] is often referred to as “pure LATE-NC” in recent literature [66, 102, 137]. The clinical diagnosis of LATE/LATE-NC and differentiation from AD/ADNC and mixed pathologies remains challenging without a molecular-specific biomarker.

There has been significant progress in identifying genetic risk factors associated with LATE-NC. Risk alleles that have been replicated are in Granulin (*GRN*), Transmembrane protein 106B (*TMEM106B*), Apolipoprotein E (*APOE*), and Sortilin related receptor 1 (*SORL1*). These genetic studies have been summarized elsewhere [102] and are not the focus of the current article. In this review, we will focus on the molecular features and clinical presentations of LATE/LATE-NC, its overlap with AD/ADNC, and the promises and challenges in developing fluid biomarkers for LATE diagnosis and differentiation from other protein-aggregating disease of dementia.

### TDP-43 proteinopathy

**Fig. 1:**
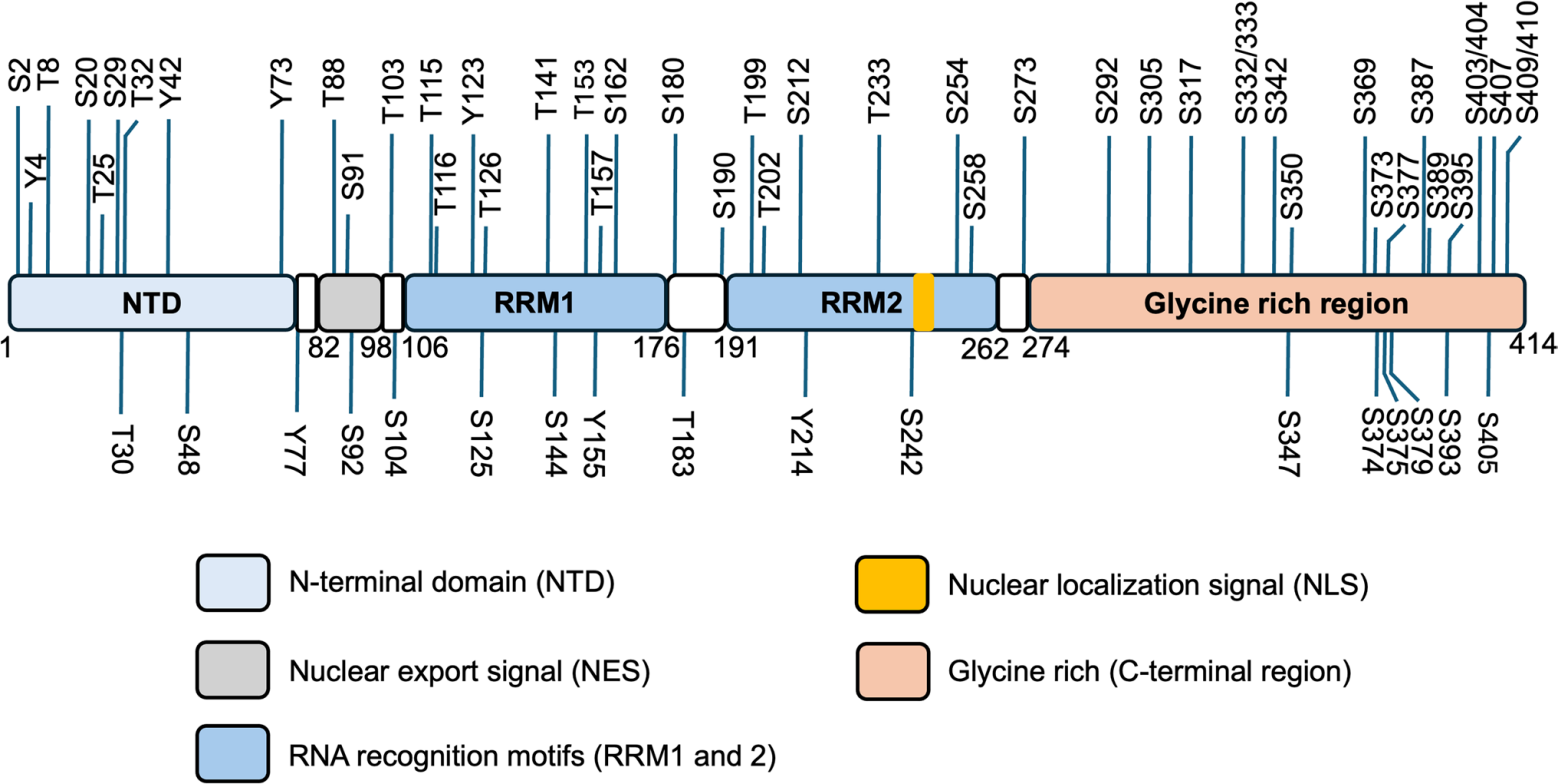
Schematic representation of TDP-43 protein domain structure and potential posttranslational modification sites. Highlighted are the TDP-43 N-terminal domain (NTD), nuclear export signal sequence (NES), RNA recognition motifs (RRM1 and RRM2), and the C-terminal region, which contains a glycine-rich (GR) domain) [125]. Possible phosphorylation sites (serine [S], threonine [T], and tyrosine [Y]) are indicated. C-terminal S409/410, S369, S379 and S403/404 have been recognized as major pathological sites in TDP-43 proteinopathies including ALS and FTLD-TDP [68]

The TDP-43 protein comprises 414 amino acids with a nuclear localization signal (NLS), two RNA-recognition motifs (RRM1, RRM2), and an intrinsically disordered glycine-rich carboxy terminal domain (Fig. 1). It regulates RNA processing pathways, mitochondrial fusion, and cellular stress [6, 33, 139]. In healthy control cells, TDP-43 is nonphosphorylated and located in the cell nuclei. TDP-43 proteinopathy is biochemically defined by the presence of relatively insoluble cytoplasmic inclusions containing ubiquitinated and abnormally phosphorylated TDP-43 [4, 74, 104, 105]. TDP-43 proteinopathy is thought to drive dual toxicity: a gain-of-function toxicity through the presence of cytoplasmic aggregates and a loss-of-function toxicity due to the depletion of normal (predominantly nuclear) TDP-43 functions [48, 68]. Among the subtypes of TDP-43 proteinopathy, LATE-NC is by far the most common [99].

Aggregated TDP-43 exhibits several posttranslational modifications including phosphorylation, acetylation, and ubiquitination [24, 42, 104]. TDP-43 phosphorylation is a major pathological hallmark feature in ALS and FTLD-TDP but not well detected in physiological conditions [42, 104]. Several C-terminal phosphorylation sites, including S409/410, S403/404, S369 and S379 (Fig. 1), have been reported most consistently in TDP-43 pathologies [42, 68, 105]. However, specific differential phosphorylation sites between LATE-NC and other TDP-43 pathologies remain to be characterized.

**Fig. 2:**
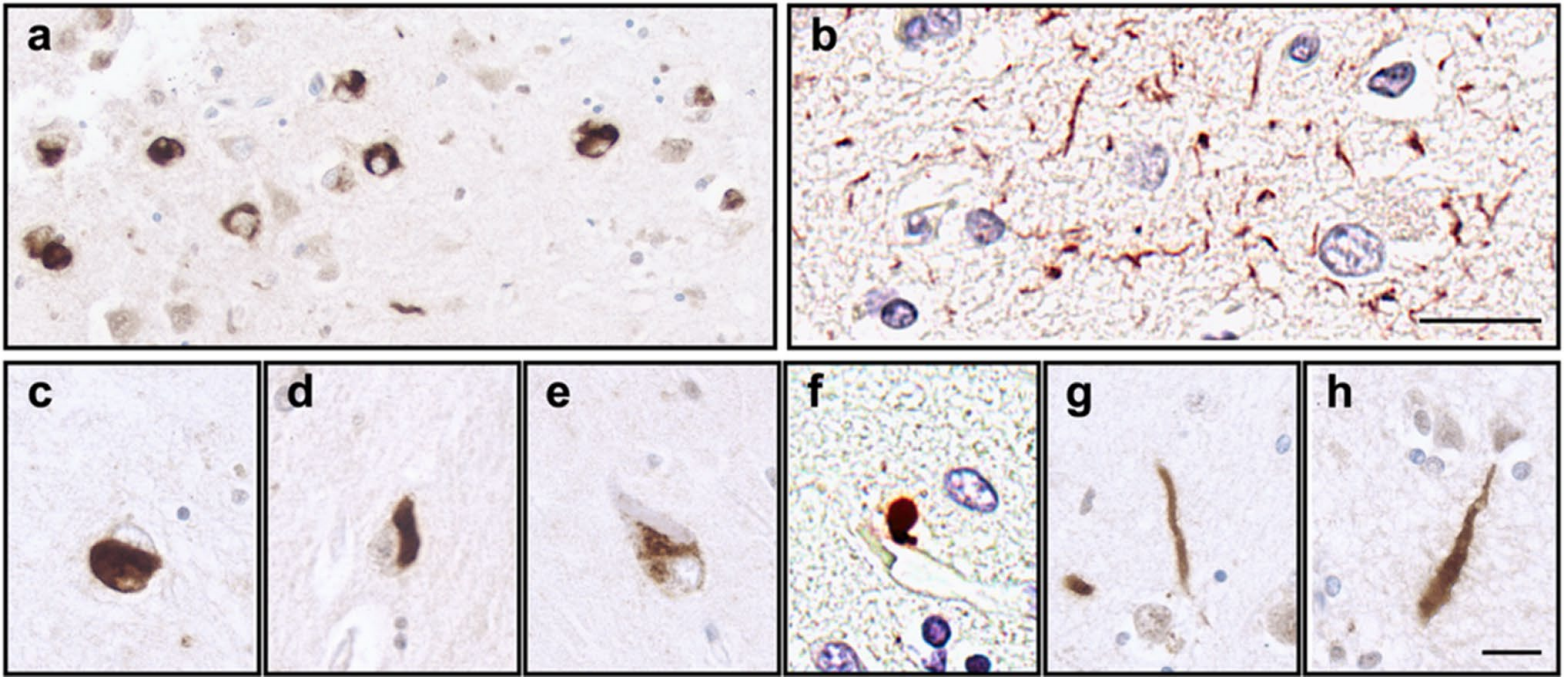
Representative images of the brain with LATE-NC, highlighting the various morphological patterns of TDP-43 proteinopathy. Some subjects and/or brain regions have predominantly neuronal cytoplasmic inclusions (**a**), while others have predominantly TDP-43 positive neurites (**b**). Common morphologies include round neuronal cytoplasmic inclusions (**c**), neurofibrillary tangle-like inclusions (**d**), granular preinclusions (**e**), perivascular “Lin bodies” (**f**), thin neurites (**g**), and thick neurites (**h**). Scale bar in **b** = 50 µm for **a**–**b**; scale bar in **h** = 10 µm for **c**–**h**

TDP-43 proteinopathy can exist in various forms and exhibit different morphological patterns [87] (Fig. 2). It is commonly found as neuronal cytoplasmic inclusions (NCI) and TDP-43 positive neurites in ALS, FTLD, and LATE-NC, but perivascular inclusions (Lin bodies) [75, 123] and astrocytic inclusions [18, 25, 84] have also been described. The different patterns of TDP-43 proteinopathy has been well-characterized in FTLD-TDP. FTLD-TDP can be divided into subtypes A to E based on the combination of TDP-43 morphologies and their distribution in cortical layers; each subtype of FTLD-TDP has its own unique clinical presentation and genetic associations [72]. For instance, FTLD-TDP type A, characterized by NCIs and dystrophic neurites in superficial cortical layers, is associated with behavioral variant frontotemporal dementia (bvFTD) or nonfluent/agrammatic primary progressive aphasia (nfPPA) and *GRN* mutations; FTLD-TDP type B, characterized by NCIs in both superficial and deep cortical layers, is associated with bvFTD with or without motor neuron disease and *C9orf72* mutations [72].

The morphological patterns of TDP-43 pathology in LATE-NC may have clinical implications and inform underlying pathogenesis as well. One classification system divided LATE-NC brains into type-α (composed of neuronal cytoplasmic inclusions, neuronal intranuclear inclusions, and TDP-43 positive neurites) and type β (composed of NFT-associated inclusions) [57]. Subjects with TDP-43 type-α are more likely to carry the *TMEM106B* risk (CC) haplotype, have hippocampal sclerosis (HS), more widespread TDP-43 pathology, and more white matter intensities on imaging, whereas those with TDP-43 type-β are more likely to have higher Braak NFT stages (Table 1) [19, 57]. More recently, four patterns of amygdala TDP-43 pathology were recognized in LATE-NC based on the morphology of TDP-43 inclusions [28]. Pattern 1 is characterized by NCIs, short neurites, and granular preinclusions, and associated with the highest age at death and most widespread TDP-43 pathology; pattern 2 is characterized by NFT-like inclusions and associated with highest levels of ADNC and enriched in APOE4 carriers; pattern 3 is characterized by round NCIs and thick neurites and associated with limbic or neocortical LBD; and, pattern 4 is characterized by subpial and white matter processes and associated with no or low ADNC (Table 1) [28]. Another study directly compared the pattern of TDP-43 proteinopathy in pure LATE-NC and ADNC + LATE-NC, and found that brains with pure LATE-NC often displayed a mesh-like neuritic TDP-43 pattern in the hippocampus (81% of pure LATE-NC vs. 18% of ADNC + LATE-NC). Moreover, while inclusions in pure LATE-NC are composed of a combination of phosphorylated and non-phosphorylated TDP-43 species, inclusions in ADNC + LATE-NC were composed predominantly of TDP-43 phosphorylated at S409/S410 [137].

**Table 1:** TDP-43 proteinopathy patterns in LATE-NC

Brain regions examined	Type/Pattern	Characteristic TDP-43 morphology	Clinical features, genetics, and neuropathological associations	References
Fourteen brain regions according to [56]	Type α	Neuronal Cytoplasmic Inclusions (NCIs), neurites	Widespread TDP-43 pathology, hippocampal sclerosis, more white matter hyperintensities on imaging, *TMEM106B* risk allele	[57]
	Type β	NFT-like inclusions	High Braak stage	
Amygdala	Pattern 1 (similar to Type α in [57])	NCIs, short neurites, granular preinclusions	Older age at death, widespread TDP-43 pathology	[28]
	Pattern 2 (similar to Type β in [57])	NFT-like inclusions	High ANDC, high Braak stage, *APOE4* carrier	
	Pattern 3	Round NCIs, thick neurites	Limbic or neocortical LBD	
	Pattern 4	Subpial and white matter neurites	Cognitively normal, no or low ADNC	

### Molecular interaction between TDP-43 and other proteinopathies

Given the frequent co-occurrence of LATE-NC with ADNC and LBD, other molecular players must also be considered when developing diagnostic biomarkers for LATE; these include amyloid-β (Aβ), α-synuclein, and tau [100, 135, 136]. TDP-43 has been implicated in the regulation of Aβ aggregation, with loss of TDP-43 linked to increased levels of soluble Aβ oligomers alongside a decrease in plaque and fibrillar deposits [70]. However, there is limited evidence to support the relationship between TDP-43 and amyloid-β synergy [136]. Some authors have also carried out biochemical and animal studies to demonstrate the potential association between TDP-43 and α-synuclein in TDP-43 pathology [45]. α-synuclein may contribute to the pathogenesis of LATE-NC by facilitating the abnormal phosphorylation and aggregation of cytoplasmic TDP-43, as demonstrated by increased phosphorylated TDP-43 inclusions in mouse brains and cultured cells following exposure to synthetic α-synuclein fibrils [106].

The interaction of tau and TDP-43 has been well-documented and is thereby described in more detail below. Accumulating evidence indicates that TDP-43 and tau pathologies often co-occur and jointly exacerbate neurodegeneration and cognitive decline [29, 87]. Immunohistochemical studies have shown that patients with both LATE-NC and ADNC have higher p-tau pathology in the posterior hippocampus and middle frontal cortex as compared to pure ADNC and cognitively normal controls (CN) [135]. Also, cells treated with frontal cortex extracts from postmortem patients with comorbid LATE-NC and ADNC conditions showed significant tau seeding as compared to pure ADNC and CN [135]. TDP-43 pathology contributes to Alzheimer’s-type dementia primarily after spreading to the hippocampus and entorhinal cortex, suggesting a synergistic interaction with tau that exacerbates disease severity [52]. This is further supported by findings that tau pathology in the limbic stage overlaps anatomically with the primary regions affected in LATE-NC, further supporting a potential intersection between tau and TDP-43 pathologies [8]. Other limbic regions such as the insular cortex and basal forebrain, known for abundant tau-immunoreactive neurofibrillary tangles, were also shown to exhibit significant accumulation of TDP-43 [56]. Autopsy results from patients with LATE-NC exhibit a hierarchical pattern of brain damage, and the concentration of tau oligomers rises with increasing levels of phosphorylated TDP-43 oligomers in the cytoplasm [87, 98]. In LATE-NC and comorbid LATE-NC + ADNC, phosphorylated tau species (p-tau) and phosphorylated TDP-43 species (pTDP-43) have been identified as major molecular players [135, 145]. There is evidence showing the coexistence of p-tau and pTDP-43 in the preclinical phase as well as in symptomatic cases in up to 80% of hippocampal neurons in AD [78, 134]. Nature of the interaction between TDP-43 and tau, however, is not well understood and may be complex as a recent study suggested that such interaction may suppress tau pathology while promoting TDP-43 pathology in AD patients [126]. Therefore, it remains to be seen if any pathogenic tau species may be directly relevant to LATE-NC pathogenesis or serve as biomarkers for LATE-NC.

### Neuropathological diagnosis and staging of LATE-NC

**Fig. 3:**
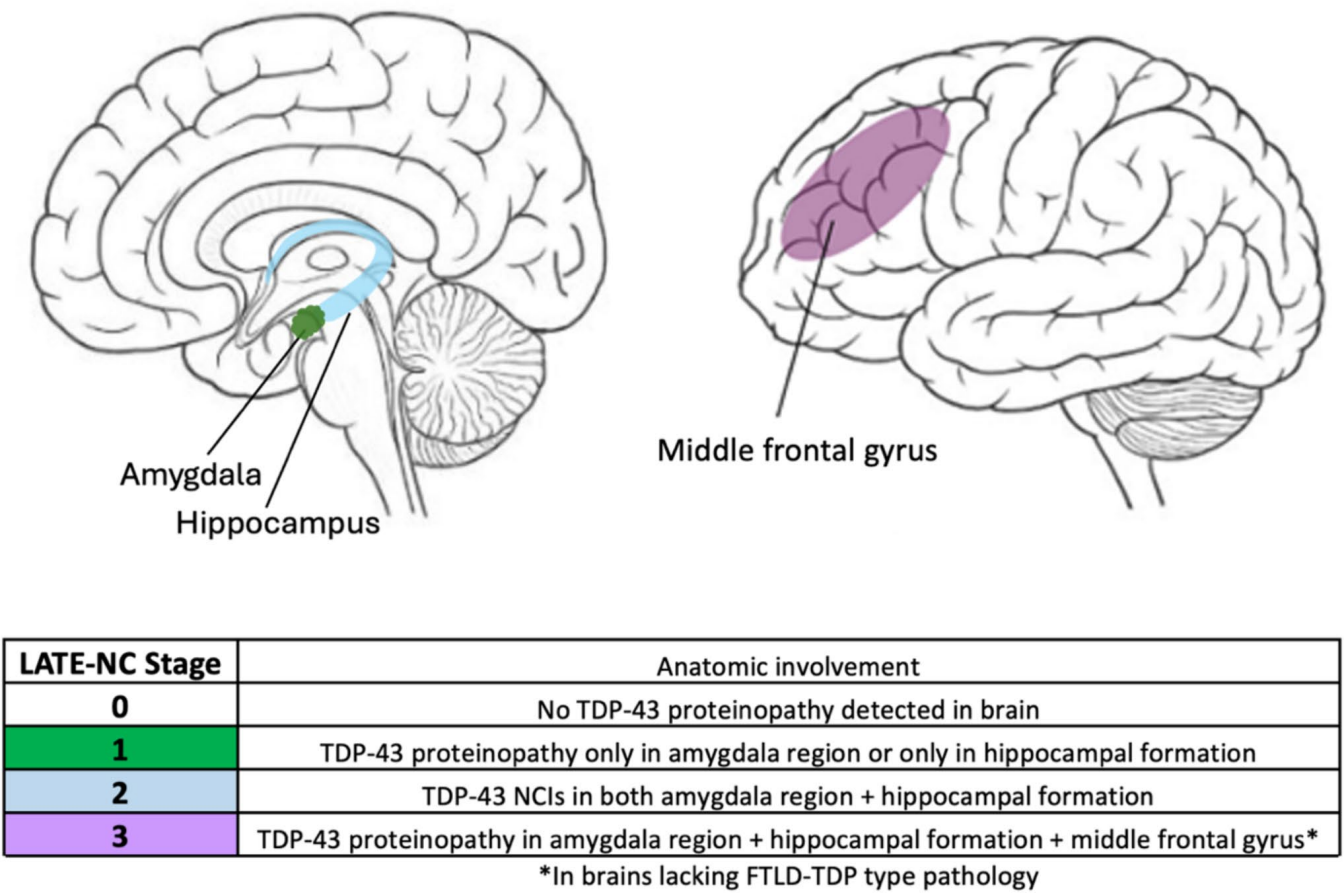
LATE-NC staging classification scheme. This figure shows a widely recognized neuropathological model used to classify the progression of LATE based on the anatomical spread of TDP-43 proteinopathy in the brain as described in the prior literature [56, 101]. The highlighted areas show TDP-43 accumulation in the part of the brain in stage 1 (amygdala), 2 (hippocampus), and 3 (middle frontal gyrus)

Autopsy results from patients with LATE-NC revealed a hierarchical pattern of brain damage, which originates from the limbic structures (amygdala and hippocampus) and extends to other regions such as the frontal cortex, consistent with neuroimaging studies [81, 98]. There are two key sets of papers that served as the bases for LATE-NC staging [54, 56, 91]. According to the consensus-based staging scheme, the amygdala is the first region to show TDP-43 pathology and is designated as stage 1, followed by spreading to the hippocampus in stage 2 and eventual progression to the middle frontal gyrus (MFG) in stage 3 (Fig. 3) [98, 101].

Presently, the existing criteria to assess LATE-NC, according to the LATE-NC consensus working group [98] is to perform TDP-43 immunohistochemical staining on at least three distinct brain regions (amygdala, hippocampus, and MFG), especially in patients with HS, clinical history of dementia and patients aged 80 years and above [98]. Although there is no age cutoff for the diagnosis of LATE-NC, and LATE-NC can occur in individuals under 80 [98], this recommendation was based on studies demonstrating that many subjects who die after 80 have cognitive decline that is out of proportion to the severity of ADNC [67, 95, 120], where LATE-NC could be a significant contributing factor, and that 60–95% of LATE-NC cases are associated with HS [3, 4, 10, 39].

One potential diagnostic challenge is distinguishing stage 3 LATE-NC from FTLD-TDP, as TDP-43 lesions resembling FTLD-TDP type A (NCIs and short neurites in superficial cortical layers) or FTLD-TDP type B (NCIs in both superficial and deep cortical layers) patterns are present in the MFG of stage 3 LATE-NC. Recent multi-institutional studies have shown that one key feature that can distinguish stage 3 LATE-NC from FTLD-TDP with high sensitivity and specificity is the density of TDP-43 lesions (NCIs or neurites) in the MFG, with higher densities (> 15 per 40X microscopic field) favoring FTLD-TDP [115, 124]. One potential pitfall is subjects with FTLD and motor neuron disease, where the density of TDP-43 lesions in the MFG can overlap with those of stage 3 LATE-NC. However, these subjects will have characteristic clinical presentations and TDP-43 pathology in motor neurons [124].

The current LATE-NC staging criteria recommends that cases with 1) FTLD-TDP type C, D, and E patterns, 2) > 15 TDP-43 lesions per 40X microscopic field, and 3) known pathogenic mutations in FTLD-TDP-related genes, such as *GRN*, *C9orf72*, and *VCP* should be classified as FTLD-TDP and not LATE-NC [101]. Further, TDP-43 lesions can be seen in many different pathologies, and a diagnosis of LATE-NC should be avoided in cases with ALS, corticobasal degeneration, chronic traumatic encephalopathy, and other rare pathologies such as Perry syndrome, Alexander disease etc. [101].

Another closely related pathology to consider in the assessment of LATE-NC is hippocampal sclerosis of aging (HS-aging). Hippocampal sclerosis (HS) is a neuropathologic phenotype that has been observed in ~ 15% of autopsied brains beyond age 85 and is strongly associated with cognitive impairment, independent of other pathologies [93]. There is no molecular-specific pathologic marker or pathognomonic feature. However, a large subset of HS is associated with LATE-NC and has been termed HS-Aging [4, 96]. From a pathologic standpoint, HS-Aging is more commonly seen in cases with more severe and widespread TDP-43 proteinopathy in LATE-NC. Although the data seem to indicate that HS-Aging is pathogenetically "downstream" of LATE-NC, it is debatable whether HS-Aging should be considered a subset of, or distinct from, LATE-NC. This is complicated by the fact that other conditions are also associated with HS-aging. A recent study from a population-based cohort showed that the positive predictive value of HS-aging on LATE-NC is modest, even in cognitively impaired individuals. This could be due to the association between HS-Aging and vascular pathologies, particularly in populations with high cardiovascular risk [107]. HS-Aging has also been identified in the clinical research context, where it has been primarily recognized via MRI scans. There is an emerging awareness that LATE-NC/HS-aging cases can have more extreme hippocampal atrophy on MRI than other conditions such as ADNC. These considerations are important because they pertain to the existing framework for clinical diagnosis of LATE-NC/HS-Aging [152].

### Clinical presentation and imaging features of LATE

The primary symptom of LATE is memory impairment [15], particularly a progressive decline in episodic memory [12, 59, 60]. Some studies have also demonstrated an association between LATE-NC and decline in semantic memory and global cognition in addition to episodic memory. Compared to pure AD, patients with pure LATE tend to live longer, show later symptom onset, and isolated memory impairment with relative preservation of other cognitive domains until much later in the disease course (Table 2) [16, 152].

**Table 2:** Comparison of LATE versus AD conditions

Features	Pure LATE-NC	Pure ADNC	ADNC + LATE-NC	References
Primary pathology	TDP-43 proteinopathy	β-amyloid plaques and tau tangles	Both ADNC and LATE-NC	[90, 98]
Brain regions affected	Limbic system predominantly	Widely distributed to include hippocampus, neocortex, and brain stem	Widespread	[90, 98, 99]
Age of onset	Often affects individuals over the age of 80	Affects individuals over 65, but early-onset AD can occur before 65	May affect younger individuals than Pure LATE-NC	[98]
Progression	Slow (but faster in LATE-NC + ADNC)	Faster than LATE-NC alone	Faster than Pure LATE-NC or pure ADNC	[12, 38, 53–55, 59, 60]
Cognitive domain affected	Episodic memory, semantic memory, and language	All	Many and often has psychoses and additional symptoms	[12, 34, 38, 59, 60, 83, 152]
Hippocampus and medial temporal lobe (MTL) atrophy	More severe hippocampal and MTL atrophy relative to the inferior temporal lobe, more severe atrophy in the anterior MTL	Uniform or more severe atrophy of the posterior MTL	Severe	[30, 31, 77, 131, 158, 159]
FDG-PET hypometabolism	More severe MTL hypometabolism relative to the inferior temporal lobe	More parietal and inferior temporal hypometabolism	Severe	[11, 14, 40]
Diagnosis	A definitive diagnosis can only be made through autopsy. Imaging can suggest probable LATE in living patients, especially those with an amyloid-negative test	Diagnosis is aided by imaging (PET scans) and CSF/plasma biomarkers, which detect amyloid and tau	Challenging at present	[51, 152]

The effects of LATE-NC on cognitive symptoms are, as a practical consideration, often influenced by other co-pathologies (Table 2). Patients with comorbid LATE-NC and ADNC show faster cognitive decline and significantly greater cognitive impairment than those with either pathology alone [16, 53–55, 59, 60]. Moreover, the combined effect of LATE-NC and ADNC leads to a broader range of cognitive and behavioral symptoms [38]. Other co-pathologies may affect the clinical presentation of LATE-NC as well. A previous study has shown that patients with LATE-NC and HS show more impairment in personal care and orientation than those without HS [39]. In contrast, some symptoms, such as urinary incontinence, appear to be associated with LATE-NC in particular [32].

There are several neuroimaging features that may be useful in the diagnosis of LATE/LATE-NC and differentiating LATE/LATE-NC from AD/ADNC. Both ADNC and LATE-NC/HS-aging cause hippocampal volume loss, but LATE-NC/HS-aging shows a stronger association with lower hippocampal volume than ADNC [30, 158, 159]. Moreover, LATE-NC is associated with more severe hippocampal and medial temporal lobe (MTL) atrophy relative to the inferior temporal lobe [131], and more severe atrophy of the anterior MTL, whereas ADNC shows a uniform pattern of MTL atrophy or a predilection for the posterior MTL [31, 77]. In addition to the well-recognized anterior–posterior gradient, a recent study demonstrated differences in the superior-inferior gradient of MTL atrophy, with LATE-NC predominantly affecting superior MTL structures (amygdala and hippocampus), and ADNC predominantly affecting inferior MTL regions (entorhinal cortex and parahippocampal cortex) [119]. Severe atrophy of the hippocampal head, anterior entorhinal cortex (ERC), and perirhinal cortex (PRC) and elevated inferior temporal lobe/MTL ratio are included as supportive neuroimaging features for the clinical diagnosis of LATE [152]. An important area for future research is to investigate the potential synergy and relative timing of tau vs. TDP-43 pathology on MTL atrophy [151]. Findings from FDG-PET are consistent with those from structural MRI, and will be described in more detail in a later section on “Molecular biomarkers of LATE”.

In addition to volume loss, structural MRI also demonstrated characteristic changes in the shape of several brain regions associated with LATE-NC, including an inward deformation of the hippocampus CA1 and subiculum, particularly in the head of hippocampus, and inward deformation of the nucleus accumbens [43, 82, 117]. Diffusion-Weighted Imaging (DWI) studies have demonstrated reduced fractional anisotropy (FA) in the limbic region with increasing TDP-43 burden, and correlation between reduced FA with impairment in episodic and semantic memory [44, 130]. Further, increased TDP-43 burden has been associated with lower R2 levels and more widespread R2 anomalies with increasing LATE-NC stage [129].

Recently, Wolk et al. proposed a set of consensus-based criteria for the clinical diagnosis of LATE based on clinical presentation and biomarkers including imaging features [152]. Due to the lack of molecular-specific, confirmatory biomarkers, the diagnosis is probabilistic and requires exclusion of other pathologies of dementia, most notably AD/ADNC. Briefly, in patient with 1) core clinical features of LATE (primary amnestic syndrome with relative sparing of other cognitive domains), 2) significant hippocampal atrophy out of proportion to global atrophy, and 3) negative amyloid biomarkers (amyloid PET, CSF Aβ42/40, CSF p-tau181/Aβ42, or CSF t-tau/Aβ42), a clinical diagnosis of *probable LATE* can be made. If amyloid biomarkers are positive, then a negative tau biomarker (tau PET or CSF p-tau181) is required for the diagnosis of *possible LATE*. If tau biomarkers are also positive, then other supportive features (such as FDG-PET with elevated inferior temporal lobe/MTL ratio, MTL/hippocampal atrophy out of proportion to tau burden on tau PET) are required for the diagnosis of *possible LATE with AD*.

Separately, Corriveau-Lecavalier et al. proposed a set of clinical criteria for limbic-predominant amnestic neurodegenerative syndrome (LANS), and patients were stratified into low, moderate, high, and highest LANS likelihood based on the number of criteria they met [27]. While the clinical entity of LANS was intended to encompass various brain pathologies that show limbic-predominance, not just LATE-NC, the diagnostic criteria incorporated many of the clinical and imaging features described above. Importantly, most patients with highest or high LANS likelihoods had autopsy confirmed LATE-NC, while most patients with moderate or low LANS likelihoods had ADNC.

A comparison of the clinical criteria for probable/possible LATE vs. LANS is the focus of a recent review [103]. At present, the sensitivity, specificity, and predictive value have yet to be established for either set of criteria, and there is no consensus-based recommendation on which criteria to use for a given patient or group of patients. Notably, the LANS criteria emphasize the utility of FDG-PET, while the probable/possible LATE criteria incorporate MRI and more fluid biomarker results, including markers of β-amyloid. It is unclear if there is long-term utility in maintaining both sets of criteria, and the criteria may evolve as more specific molecular markers for LATE-NC become available.

### Molecular biomarkers of LATE

Unlike extensively investigated imaging and biofluid biomarkers for other neurodegenerative diseases, such as AD, the field of biomarker research for LATE is still in its early stages [34]. In the absence of clinically validated molecular-specific biomarkers, the diagnosis of LATE remains challenging. In this section, we describe recent efforts to develop molecular biomarkers for TDP-43 proteinopathies. While the focus of this review is on LATE/LATE-NC, we also include biomarkers developed for other TDP-43 proteinopathies such as ALS and FTLD.

**Fig. 4:**
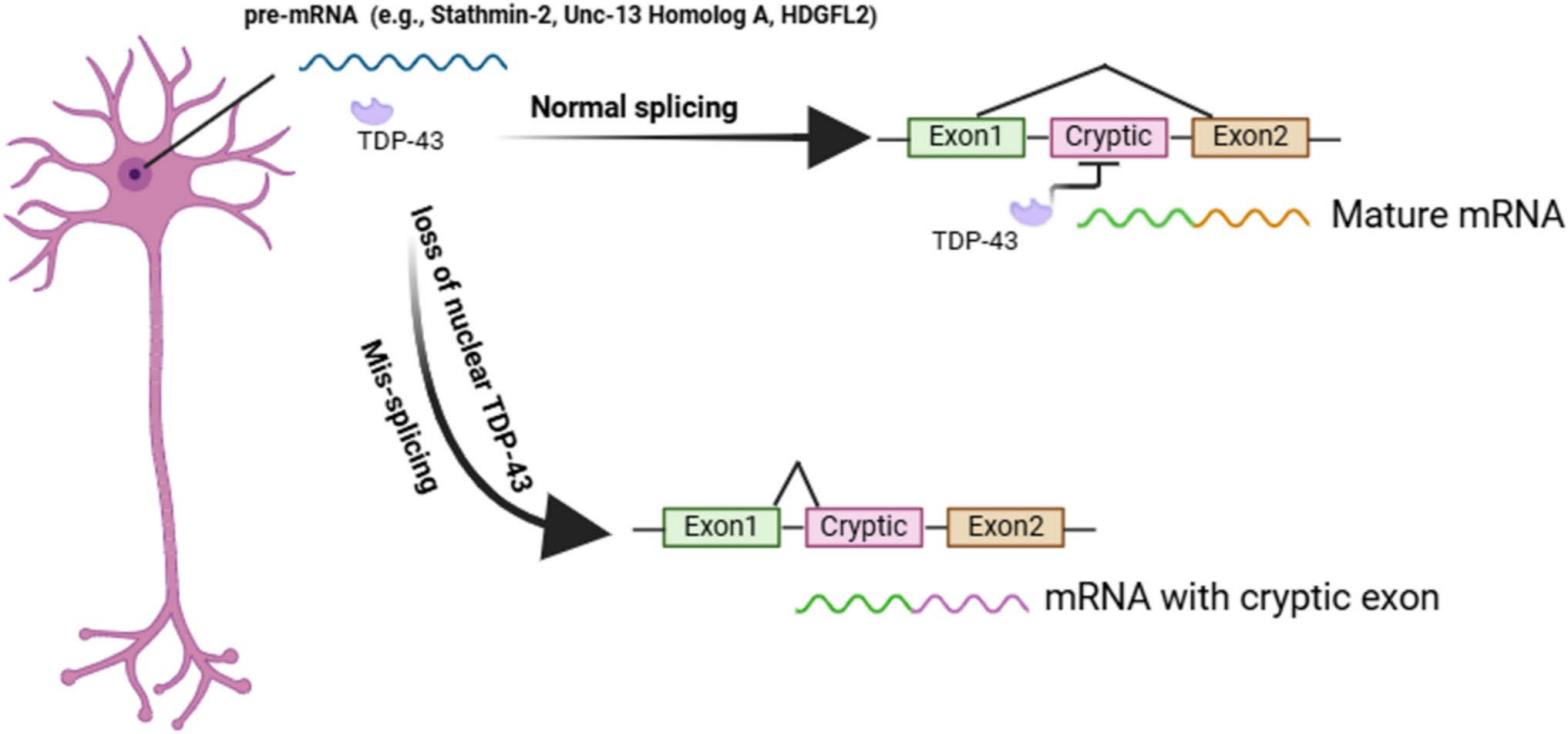
Schematic representation of how aberrant TDP-43 protein leads to cryptic RNAs/exons which may serve as biomarkers in TDP-43 proteinopathies. The processes of normal and alternative splicing of pre-mRNA involving TDP-43 are illustrated. It shows how mature mRNA is produced through standard splicing, retaining specific exons, while also depicting the occurrence of cryptic exons, resulting from mRNA mis-splicing. The role of TDP-43 in regulating these splicing events is emphasized, highlighting its influence on mRNA processing

1. **Imaging**
2. Fluorodeoxyglucose positron emission tomography (FDG-PET)—LATE-NC is associated with more severe MTL hypometabolism relative to the inferior temporal lobe (temporo-limbic pattern), whereas ADNC is associated with more severe hypometabolism in the parietal and inferior temporal lobes (temporoparietal pattern) [11, 14, 40]. Limbic hypometabolism in the absence of cortical neurodegeneration on FDG-PET imaging has been proposed as one of the criteria of limbic-predominant amnestic neurodegenerative syndrome (LANS), a clinical syndrome that shows strong correlation with LATE-NC [27]. However, a recent study showed that FDG-PET has only moderate predictive accuracy in identifying brain regions associated with LATE-NC [71]. Furthermore, pure LATE patients will have negative tau/amyloid beta PET results, while those with AD or AD comorbid with LATE will have a positive tau/amyloid beta PET [152].
3. TDP-43 PET—Currently, there are no clinically-approved PET tracers for TDP-43. Recently, two small molecule radiopharmaceuticals, [^18^F]ACI-19278 and [^18^F]ACI-19626, have been developed as TDP-43 PET tracers [142]. Both ligands show high binding affinity to aggregated, but not soluble, TDP-43 in brain tissue from patients with FTLD-TDP, ALS, and LATE, and show excellent selectivity for TDP-43 over other protein aggregates, including amyloid-β, tau and α-synuclein. Of note, both ligands show pharmacokinetic profiles suitable for PET imaging in vivo, making them promising candidates for translation into clinical use in a subset of TDP-43 proteinopathies including LATE [49].
4. **Biofluids**
5. Plasma TDP-43 levels—Blood-based extracellular vesicles (EVs), specifically measuring TDP-43 concentration in astrocyte-derived EVs (ADEVs), showed promising capability to differentiate LATE-NC (+) from LATE-NC (-) subjects (Table 3) [150]. However, methods for EV isolation are not widely available for clinical use. Plasma TDP-43 levels have been shown to correlate with neurodegeneration in specific limbic system brain regions on imaging [9]. However, detecting pathological TDP-43 in biofluids such as CSF and serum remains challenging due to antibody cross-reactivity with both normal and pathological forms, aggregation affecting solubility, and its widespread expression throughout the body [79]. Recent advancements in immunoassay technologies have markedly enhanced the accuracy and sensitivity of TDP-43 quantification in these biofluids [5, 85]. A new study measuring plasma TDP-43 levels with highly sensitive Nucleic Acid Linked Immuno-Sandwich Assay (NULISA) showed promising results in detecting patients with advanced LATE and comorbid AD, but not patients with pure LATE [147]. Whereas only a limited number of studies have explored the utility of biofluid TDP-43 levels in LATE, plasma and/or CSF TDP-43 levels (in a few cases, also pTDP-43 levels) showed significant elevation in ALS patients compared to healthy control in multiple studies (Table 3).
6. Cryptic RNAs—Biomarkers reflecting TDP-43 dysfunction can be detectable in biofluids. Although most of these biomarkers have been investigated in the context of FTLD-TDP and ALS, given the shared pathological feature of progressive TDP-43 proteinopathy, it is reasonable to consider that proteins identified in these conditions as discussed in Table 4 might also hold relevance for LATE [2, 34]. TDP-43 normally binds to certain RNA transcripts (for example, Stathmin-2, Unc-13 Homolog A, hepatoma-derived growth factor like protein 2 (HDGFL2), and DNA Polymerase Delta Interacting Protein 3) to prevent the inclusion of cryptic exons. However, mislocalized or dysfunctional TDP-43 fails to regulate cryptic exon splicing, leading to abnormal RNA species (Fig. 4), which is particularly evident in the hippocampus of patients with both pure LATE-NC and LATE-NC with comorbid ADNC. For instance, loss of TDP-43 function leads to the exposure of a cryptic polyadenylation site in STMN2, a critical gene for axon maintenance and repair, causing a buildup of truncated STMN2 mRNA and a reduction in its normal full-length form [69, 86, 109]. tSTMN2 was elevated in RNA extracted from frontal cortex of FTLD-TDP compared with controls (p < 0.005) and the rare tauopathy progressive supranuclear palsy (PSP) [109]. Relative qPCR expression of several cryptic exons in the hippocampus also revealed truncated stathmin-2 (tSTMN2) as the most significant cryptic RNA specific for LATE-NC (Table 4). tSTMN2 was able to distinguish pure LATE-NC from healthy controls and ADNC cases [23, 138]. Another study showed that exclusion of TDP-43 from the nucleus in brains with LATE-NC is associated with expression of a cryptic exon in HDGFL2 and may precede the appearance of TDP-43 positive neuronal cytoplasmic inclusions by a decade [21]. Cryptic HDGFL2 has been detected in CSF at significantly higher levels in TDP-43 proteinopathy in ALS compared with controls and has been proposed as a potential diagnostic biomarker for ALS [50]. Higher levels of cryptic exons including tSTMN2 in hippocampal tissues from patients with LATE-NC and comorbid ADNC + LATE-NC in comparison to those from ADNC have been reported in a recent mass spectrometry-based study (Table 4) [138]. Detection of cryptic exons in CSF or plasma from patients with LATE/LATE-NC has not been reported yet.
7. Emerging neuronal biomarkers—Loss of nuclear TDP-43 has been linked to the dysregulation of specific neuronal transcripts, including synaptic neuronal pentraxin 2 (NPTX2) mRNA [46]. Studies have demonstrated that in neurons overexpressing TDP-43 and in postmortem brains exhibiting TDP-43 proteinopathy, there is a significant upregulation of NPTX2 mRNA. This upregulation results from the loss of TDP-43’s ability to bind to the GU-rich region of NPTX2 mRNA [46, 133]. The consistent association between nuclear TDP-43 loss and increased NPTX2 mRNA levels suggests that NPTX2 may serve as a potential biomarker for TDP-43 proteinopathies [26]. Other potential biomarkers include proteins involved in calcium signaling and synaptic communication, such as calsyntenin-1 and neurexin-2a, which have been observed in cerebrospinal fluid and correlate negatively with TDP-43 burden [20]. These neuronal markers may offer insight into TDP-43-related neurodegeneration and could potentially serve as indicators of disease progression in LATE.
8. Biofluid proteomics—Assessment of proteomes of autopsy-confirmed LATE-NC and non-LATE-NC CSF did not show a potential proteomic fingerprint that can distinguish the two groups. However, results from western blot analysis conducted for four proteins, namely RBP4, MIF, IGHG3, and ITM2 B, showed that RBP4 levels were higher in LATE-NC patients in comparison to patients without LATE-NC (p = 0.03) [37].

**Table 3:** Studies utilizing antibody-based methods to detect abnormal TDP-43 in biofluid samples for the diagnosis of LATE-NC or other TDP-43 proteinopathies

Biofluids	Technique	Pathological Groups	Results	Reference
LATE-NC				
Plasma ADEVs	ELISA	LATE-NC (n = 22) Control groups (included healthy controls, AD MCI, and AD dementia with diagnosis other than LATE-NC) (n = 42)	TDP-43 level was elevated in plasma ADEVs from autopsy confirmed LATE-NC patients with or without ADNC comorbidity as compared to control subjects (p < 0.0001) TDP-43 levels in EV-depleted plasma were not significantly different between LATE-NC(+) versus LATE-NC(-) subjects	[150]
Plasma	SIMOA	Stage 2 LATE-NC (n = 21), Stage 3 LATE-NC (n = 5), and Non-LATE-NC cases (n = 22)	This preliminary report found that TDP-43 levels were higher in LATE-NC(+) relative to LATE-NC(-) cases, however, the elevation was not statistically significant. The mean concentration of TDP-43 tends to increase with increased staging of LATE-NC	[127]
Plasma	NULISA	Stages 0 and 1 LATE-NC (n = 50), Stages 2 and 3 LATE-NC (n = 50)	Plasma TDP-43 level was significantly elevated in individuals with advanced LATE-NC (stages 2 and 3) compared to controls (stage 0 and 1; p = 8.8 × 10^–3^)	[147]
Other TDP-43 Proteinopathies				
CSF	ELISA	ALS (*n* = 30) CN (*n* = 29)	TDP-43 concentrations in the CSF were significantly higher in the ALS group compared to age-matched control subjects (p = 0.023)	[63]
Plasma	ELISA	ALS (*n* = 219) CN (*n* = 100)	Elevated TDP-43 plasma levels in ALS patients as compared to CN subjects (p = 0.023)	[141]
Plasma	ELISA	FTLD (*C9orf72* expansions; n = 10) FTLD (*GRN* mutations; n = 5) CN (n = 22)	Plasma pTDP-43 levels were elevated in FTD patients with C9orf72 expansions or GRN mutations compared to healthy controls (p < 0.05)	[128]
CSF	ELISA	ALS (n = 21) FTLD (n = 69)	ALS patients showed increased CSF TDP-43 levels compared to FTLD patients (p = 0.001)	[58]
CSF / Plasma	SIMOA	Discovery Cohort: ALS (n = 29) Non-neurodegenerative control (n = 29) Validation Cohort: ALS (n = 46) Non-ALS neuromuscular control (n = 46)	For the discovery cohort, ALS patients showed increased TDP-43 levels compared to control subjects with CSF samples (p < 0.0001) and plasma samples (p = 0.0035). For the validation cohort, ALS patients showed increased TDP-43 levels compared to non-ALS neuromuscular controls with CSF samples (p = 0.0087), but not with plasma samples	[64]
Plasma	ELISA	ALS (*n* = 69) CN (*n* = 59)	Plasma levels of TDP-43 and pTDP-43 were significantly elevated in ALS patients compared to control subjects (p < 0.001)	[110]
Plasma	SIMOA	FTLD (*n* = 254) CN (*n* = 105)	Serum total TDP-43 levels were significantly reduced in the FTLD group compared to healthy controls (p = 0.034)	[65]
Plasma	SIMOA	ALS (*n* = 17) CN (*n* = 17)	Plasma TDP-43 levels were higher in ALS as compared to CN groups (p = 0.03)	[85]
Plasma	MSD	ALS (*n* = 101) CN (*n* = 115)	There is a significant decrease in full-length TDP-43 levels in the plasma of ALS patients compared to healthy controls (p = 0.001)	[5]

*ADEV* astrocytes derived extracellular vesicles, *MCI* mild cognitive impairment, *MSD* Meso Scale Discovery, *NULISA* nucleic acid linked immuno-sandwich assay, *pTDP*-*43* phosphorylated TDP-43, *SIMOA* Single Molecule Array

**Table 4:** Summary of cryptic RNA and other potential biomarkers studied in LATE-NC and other TDP-43 proteinopathies

Biomarker	Sample used	Method	Pathological group	Findings	Reference
LATE-NC					
Cryptic exons: tSTMN2, UNC13A, ELAVL3, KALRN, ARHGAP32, CAMK2B	Hippocampal tissue	TMT mass spectrometry	LATE-NC (n = 10), ADNC (n = 25), ADNC + LATE-NC (n = 35), CN (n = 21)	Higher levels of tSTMN2 CE and KALRN CE in LATE-NC as compared to CN (p < 0.05) in both cases. Higher levels of STMN2 CE, ELAVL3 CE, KALRN CE, ARHGAP32 CE, CAMK2B CE in ADNC + LATE-NC as compared to CN (p < 0.001) in all cases. Higher levels of tSTMN2 CE, UNC13A CE, ELAVL3 CE, ARHGAP32 CE and CAMK2B CE in ADNC + LATE-NC in comparison to AD (p < 0.001)	[138]
Other TDP-43 Proteinopathies					
tSTMN2 RNA	Frontal cortex	Nanostring PlexSet platform	FTLD-TDP (n = 238), PSP (n = 41), controls (n = 33)	Higher STMN2 level is observed in FTLD -TDP as compared to PSP (p < 0.001) and controls (p < 0.005)	[109]
Chitotriosidase (Chit 1)	CSF	ELISA	ALS (n = 118), disease control (DC; n = 17), healthy control (HC; n = 24)	Chit-1 level is higher in ALS as compared to DC (p < 0.01) and HC (p < 0.0001)	[143]
Chitinase-3-like protein 1 (CHI3L1)	CSF	ELISA	ALS (n = 118), disease control (DC; n = 17), healthy control (HC; n = 24)	CHI3L1 level is higher in ALS as compared to HC (p < 0.05)	[143]
C-terminal to N-terminal TDP-43 peptide ratio	Motor and prefrontal cortex urea fractions	Mass spectrometry	ALS (n = 15), AD (n = 8), PD (n = 8), Control (n = 8)	Significantly elevated levels of C-terminal to N-terminal TDP-43 peptide ratios in ALS cortices compared to AD, PD, and controls (p < 0.0001 respectively)	[35]
Cryptic exons: CAMK2B, CDK7, ARHGAP32, ACTL6B, HDGFL2, SYT7	Frontal cortex	RT-PCR	FTLD-TDP (n = 89), Control (n = 27)	Levels of cryptic RNAs higher in FTLD as compared to CN (p ≤ 0.0001) in all cases	[122]
HDGFL2-CE	Amygdala and frontal cortex	MSD immunoassay	FTLD-TDP (n = 67), AD TDP (n = 70), AD no TDP (n = 27), Control (n = 26)	Levels of HDGFL2-CE is higher in FTLD-TDP as compared to CN in both brain regions (p < 0.0001). Levels of HDGFL2-CE is higher in AD cases with TDP-43 pathology (AD TDP) as compared to CN in amygdala (p < 0.0001), but not in frontal cortex	[17]
HDGFL2-CE	CSF	MSD immunoassay	Sporadic ALS (n = 44), symptomatic C9orf72 (n = 76), presymptomatic C9orf72 (n = 81), controls (n = 66)	Levels of HDGFL2-CE are higher in ALS as well as symptomatic and presymptomatic C9orf72 mutation carriers as compared to controls (p < 0.0001, p < 0.001, and p < 0.01 respectively)	[50]

*CE* cryptic exon, *HDGFL2* hepatoma-derived growth factor-like protein 2, *MSD* Meso Scale Discovery, *PD* Parkinson’s disease, *PSP* progressive supranuclear palsy, *TMT* Tandem Mass Tag, *tSTMN2* truncated stathmin-2

Currently there are no robust fluid biomarkers available to differentiate pure LATE from CN individuals or those with AD and comorbid AD + LATE. Challenges in developing LATE-specific TDP-43 biomarkers include factors such as the lower burdens of TDP-43 pathology observed in LATE-NC compared to FTLD-TDP or ALS, intracellular location of the protein, and possible different TDP-43 fibril structural fold in LATE-NC versus in other TDP-43 proteinopathies [7]. However, recent technological advances in ultrasensitive detection of misfolded proteins may provide significant opportunities for detecting disease-specific TDP-43 species in biofluids such as CSF and/or plasma. These sensitive detection technologies, including Single Molecule Array (SIMOA) [111, 112, 156], misfolded protein templated seeding amplification assays, such as Real-Time Quake-Induced Conversion (RT-QuIC) [144, 146, 154, 160], and Nucleic acid Linked Immuno-Sandwich Assay (NULISA) [36, 147], have shown strong promises in detection of misfolded proteins in biospecimens of other neurodegenerative diseases such as AD and prion disease. Novel biomarker discoveries may also be facilitated by identification of high-performance antibodies recognizing pathogenic TDP-43 species in disease samples [161]. Furthermore, as exemplified by phospho-tau AD biomarkers discovery and development in the field [62, 148], advanced, quantitative mass spectrometric mapping of posttranslational modifications, such as site-specific phosphorylation, in TDP-43 from LATE patients in comparison with those from normal controls may enable novel pTDP-43 epitope discoveries. Additionally, neuronal biomarkers identified in other TDP-43 proteinopathies may also be evaluated to identify LATE-specific fluid biomarkers. Furthermore, given the emerging evidence of interaction between TDP-43 and tau pathology in LATE, and the established reliability of phosphorylated tau (p-tau) as a biofluid biomarker in AD [62, 155], the potential utility of p-tau in the diagnosis and differentiation of LATE from AD or comorbid AD + LATE deserves further exploration.

## Data Availability

No datasets were generated or analysed during the current study.
